# Bow-tie architectures in biological and artificial neural networks: Implications for network evolution and assay design

**DOI:** 10.1016/j.isci.2023.106041

**Published:** 2023-01-25

**Authors:** Seth Hilliard, Karen Mosoyan, Sergio Branciamore, Grigoriy Gogoshin, Alvin Zhang, Diana L. Simons, Russell C. Rockne, Peter P. Lee, Andrei S. Rodin

**Affiliations:** 1 Department of Computational and Quantitative Medicine, Beckman Research Institute, City of Hope National Medical Center, 1500 East Duarte Road, Duarte, CA 91010, USA; 2 Department of Immuno-Oncology, Beckman Research Institute, City of Hope National Medical Center, 1500 East Duarte Road, Duarte, CA 91010, USA

**Keywords:** Molecular network, Neural networks, Artificial intelligence

## Abstract

Modern artificial neural networks (ANNs) have long been designed on foundations of mathematics as opposed to their original foundations of biomimicry. However, the structure and function of these modern ANNs are often analogous to real-life biological networks. We propose that the ubiquitous information-theoretic principles underlying the development of ANNs are similar to the principles guiding the macro-evolution of biological networks and that insights gained from one field can be applied to the other. We generate hypotheses on the bow-tie network structure of the Janus kinase - signal transducers and activators of transcription (JAK-STAT) pathway, additionally informed by the evolutionary considerations, and carry out ANN simulation experiments to demonstrate that an increase in the network’s input and output complexity does not necessarily require a more complex intermediate layer. This observation should guide novel biomarker discovery—namely, to prioritize sections of the biological networks in which information is most compressed as opposed to biomarkers representing the periphery of the network.

## Highlights

• ANNs and biological networks have similar underlying information-theoretic principles
• Naturally evolved and artificially trained networks result in similar structures
• These ubiquitous structures should be targeted for biomarker discovery
• Modern computer science and evolutionary biology can benefit from others' insights

## Graphical abstract

**Figure:**
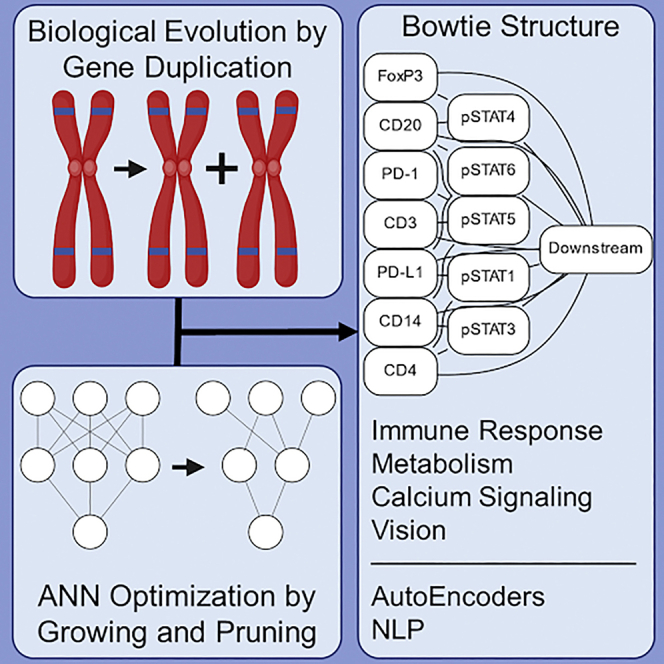

## Introduction

The foundation of the artificial neural network (ANN) was first formulated by simulating biological neural events as propositional logic.^1^ The origin of weight adjustment was fashioned after the Hebb’s rule, which describes how connections between neuronal cells increase in strength after persistent and causal stimulation.^2^^,^^3^ This pattern of neural network biomimicry served as the primary driver in much of early ANN development.^4^^,^^5^^,^^6^ However, for the last several decades, the ANN breakthroughs have not been driven by biology—for instance, backpropagation was a major development in artificial intelligence research, yet there was no biological inspiration for such an idea at the time.^7^ Still, debates over the similarities between ANNs and actual neural functions have continued into recent years.^8^ Here, we propose that certain modern ANN practices, which developed on non-biological foundations, are reflective of ubiquitous information transfer properties that govern not only the human brain function but also the entirety of biological networks’ evolution.

**Figure 1:**
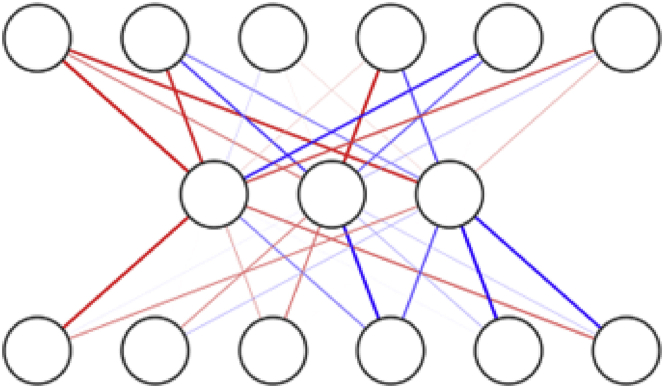
Bow-tie structure An example of an arbitrary bow-tie structure. Line opacity represents the strength of the connection between two given nodes. Some bow-tie structures might include further complexity in the form of agonist/antagonist relationships, represented by the red and blue connections. The general structure of all bow ties is the same—fewer connective nodes in the middle layer(s) than in the outside layers.

Historically, science concepts generally flow from natural sciences to social sciences. However, centrality (which is the idea of measuring connectedness in a graph) has flowed “backward,” from the complexities of social science to biology.^9^ Studies on the centrality and related information transfer concepts have led to the discovery of consistent patterns, one of which is the bow-tie structure (see Figure 1).

There are a multitude of well-known examples of biological bow-tie structures spanning across different lineages and various biological systems, from metabolism to cell receptor structures,^10^^,^^11^^,^^12^^,^^13^^,^^14^^,^^15^^,^^16^^,^^17^^,^^18^^,^^19^^,^^20^^,^^21^^,^^22^ suggesting evolution toward an (at least temporarily) optimal evolutionary state. Bow ties are also being studied outside of biology, as part of complex systems science.^23^ As in the biological domain, there are many non-biological examples where the bow-tie structure is shown to be the most optimal way of organizing complex systems.^24^^,^^25^^,^^26^^,^^27^ In general, the bow-tie architecture is seemingly representative of universal organizational principles of complex networks and suggests a general rule for efficient information transfer. Determining such principles underlying a biological function is necessary for understanding even just one process end to end.^28^^,^^29^

### Network growing and pruning

Modern machine learning (ML) practice often demonstrates “overgrown” ANNs achieving high accuracy, thus defying the “classical” ML paradigm of the U-shaped bias-variance tradeoff. This phenomenon is observed as the “double-descent curve”^30^—highly overparameterized models are expected to overfit the training data yet still result in incredibly high test accuracies.

**Figure 2:**
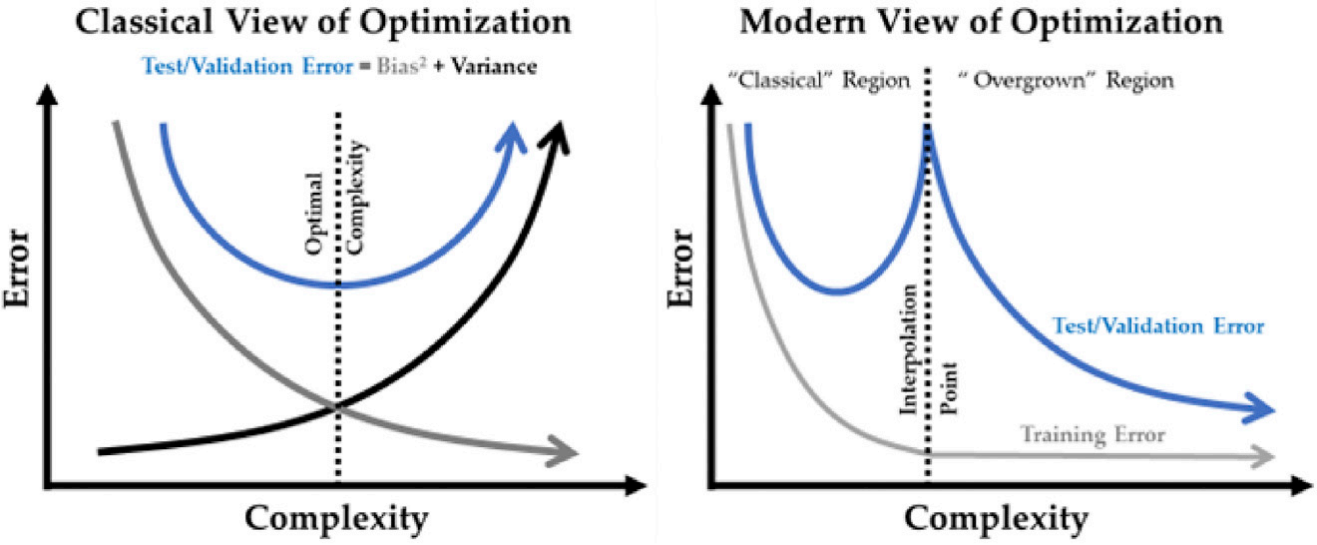
The double-descent phenomenon The left panel illustrates the “classical” view of how to optimize a neural network while the right panel illustrates the modern view (which also includes a simplified version of the left figure in the space defined as the “classical” region). Notably, the test/validation error is still high at the start of the “overgrown” region and only starts to decrease after the complexity increases dramatically.

An overparameterized ANN is referred to as an “overgrown” ANN, in which the practitioners make the number of layers, the width of the layers, and the connectedness of the layers as large as is computationally possible. Conventional ML practice would have the model optimized toward the lowest error in the “classical” region, whereas modern ML practice shows that this does not always produce the highest possible generalization accuracies (see Figure 2).

**Figure 3:**
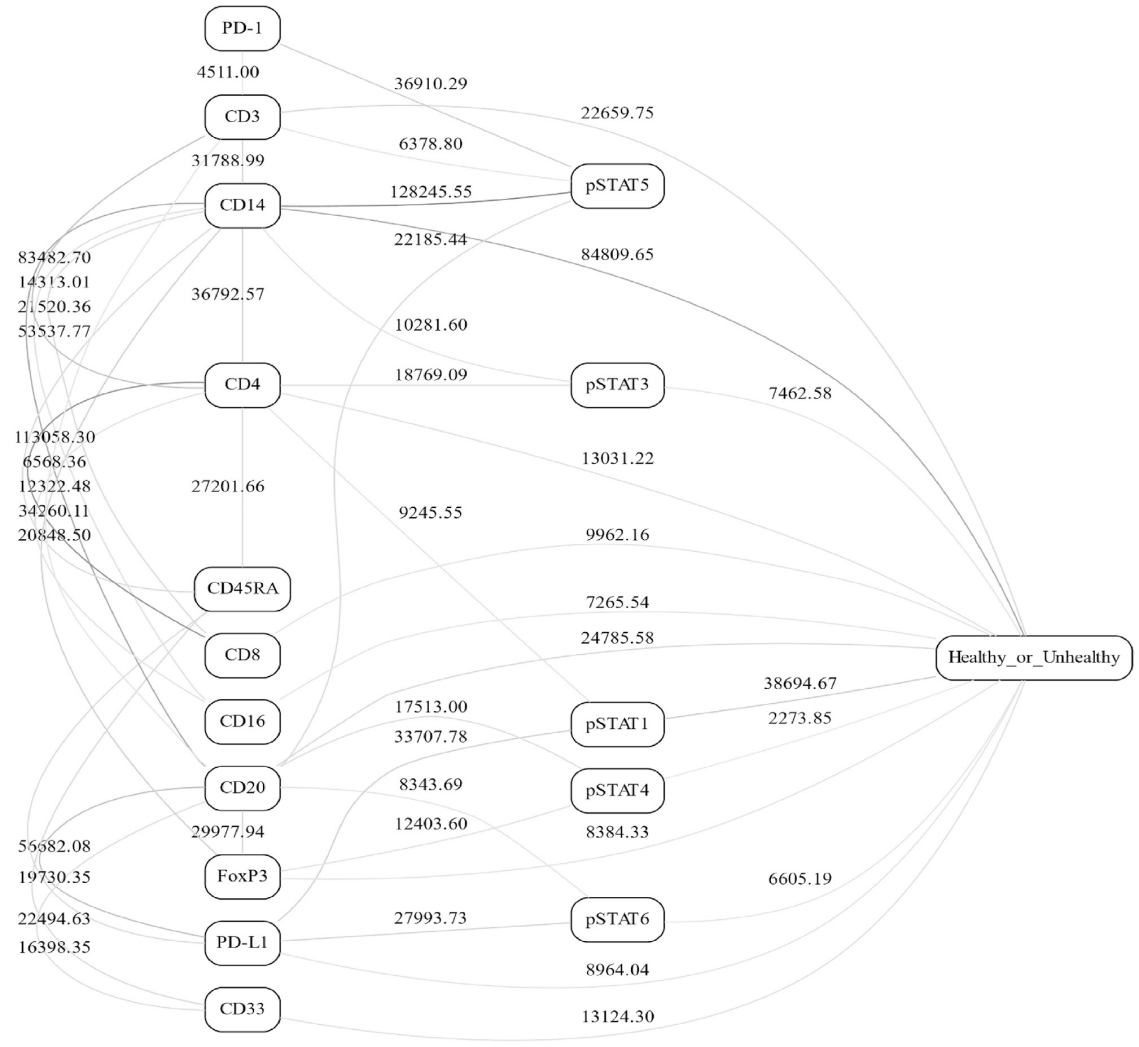
Bayesian network of PBMCs in breast cancer patients and healthy controls stimulated with various cytokines The number next to the edge and edge opacity indicate dependency strength. Edge strengths are not bounded. The nodes are “healthy_or_unhealthy”, whether the cell came from a healthy control or a patient with cancer. PBMC, peripheral blood mononuclear cells; pSTATx, phosphorylated STATs. All the remaining nodes are cell surface markers. See text (method details) for further details.

A vast (and only a vast) increase in model complexity often results in the highest generalization accuracy. However, these large models tend to be cumbersome and energy-inefficient. To combat this, the modern practice utilizes a “pruning” technique, in which unimportant artificial neurons (or connections) are removed after the model has been trained. This results in a substantial reduction of model size while retaining similar or occasionally even higher accuracies (see Figure 3 in the study by Frankle and Carbin^31^). Thus, an “overgrowth”-“pruning” regime is established. The development of the human brain seems to follow similar principles—infants produce millions of connections during their initial brain development and then slowly start losing connections over time through a process called synaptic pruning.^32^^,^^33^ Interestingly, this also seems to apply to the idea that higher accuracies might be achieved through certain levels of pruning: Decreased synaptic pruning has been associated with autism spectrum disorder,^34^ while increased synaptic pruning has been associated with schizophrenia^35^—implying an optimal level of synaptic pruning is required for achieving the biological equivalent of “high accuracies,” regardless of the optimal energy consumption.

During the pruning process, redundant nodes are rarely found in the input or output layers, meaning connections and nodes are typically removed from the middle layers and result in a bow-tie structure. Indeed, many architectures are built with a bow-tie structure *a priori*, such as in any kind of encoder-decoder approach.^36^^,^^37^

The mathematical treatment of bow-tie structures’ evolution, in biological context, has been presented by Friedlander et al.^24^ Briefly, when the number of inputs exceeds the number of outputs in a system (or when an equal inputs-outputs system is subjected to even a minimal noise, as is the case with typical biological systems), Friedlander et al. demonstrate that the system spontaneously evolves toward a bow-tie structure. These conditions are almost always met within the biological network systems—the noisy input space of all possible pathogens “fed” into the immune system, for instance, dwarfs the variety of possible responses to these pathogens. Therefore, while the previous biological example was of the process of brain development—which spans the course of a single organism’s life—we suggest that the information transfer properties underlying this development also guide macroevolutionary processes that span the course of the development of entire species. Below, we show evidence of a bow-tie structure existing within a key immune system pathway and simulate ANN models of a similar structure and dimensionality to demonstrate how it could have evolved via a “growing and pruning” paradigm.

## Results

### Bow-tie structure of the JAK-STAT signaling pathway

Our group has been pursuing Bayesian network (BN) modeling analysis of various immune network systems,^38^ one of which is the Janus kinase - signal transducers and activators of transcription (JAK-STAT) signaling pathway.^39^ The JAK-STAT pathway is widely conserved across species and is considered a core signaling pathway in health and cancer.^40^^,^^41^ Upon performing BN analyses (see method details/Bayesian network (BN) modeling) on peripheral blood mononuclear cells (PBMCs) collected from breast cancer patients and healthy controls that were stimulated by varying dosages of cytokines (see method details/biological data generation), we found information flow that appears similar in nature to a bow-tie structure (Figure 3). In this multimodal BN, while there are connections between surface-cell receptors and connections that bypass the phosphorylated STATs, we see narrowing/streamlining of connection structure as one proceeds from external cell receptors toward the downstream outcome (i.e., whether cells are dysfunctional or not).

The network shown in Figure 3 is not necessarily representative of a normal-functioning immune system. It is constructed from a collection of many types of PBMCs (each cell being a single data point used for the recovery of the BN) obtained from both healthy donors and cancer patients. This largely explains the connections between various cell surface receptors, for example, there is a strong (negative) correlation between CD8 and CD4 because these are not typically found on the same cell/data point. The downstream outcome as encoded in the binary “healthy_or_unhealthy” node has a multitude of components and confounding variables not measured here—indeed, the notion of “healthy or unhealthy” is an encapsulation of a large number of responses and events (e.g., cell classification and differentiation) within the cell nucleus, condensed into a single variable. While BNs are not directly comparable to ANNs, the overall concept of information propagation is a key factor in both types of networks (probabilistic inference in BNs, feedforward propagation with backpropagation learning in ANNs). BNs are more suitable for modeling the mechanistic biological networks from the actual data, while ANNs are more convenient for simulation experiments on varying types of network architectures. With any network representation, even in this limited example, we see the first half of a bow-tie structure: a large number of connections between the surface-cell receptors that then feed into the comparatively compact STAT system, followed by a connection to a representation of many downstream actions.

If the bow ties are a universally optimal information-processing scheme, are there any known JAK-STAT evolutionary parallels to ANN development that could support the notion that JAK-STAT is an evolutionary optimized bow-tie structure?

### JAK-STAT signaling pathway evolution by gene duplication

Hundreds of millions of years ago, several whole-genome duplication (WGD) events and tandem gene duplication events occurred, which marked splits in the phylogenetic tree of ancestral vertebrates.^42^ These processes are generally responsible for making replicates, which then occasionally mutate into new functionalities.^43^ It is believed that adaptive immunity emerged from such duplication processes, which caused either neofunctionalization or subfunctionalization of the duplicate STATs.^44^^,^^45^^,^^46^ We suggest that the duplication events, with WGDs first and foremost, are the equivalent of creating an overgrown ANN. In parallel, upstream cytokine receptors were recruited as additional inputs for the JAK-STAT signaling pathway. Then, pathogens served as the selective force for JAK-STAT gene retention.^42^^,^^45^ This is analogous to the process of optimizing “weights” to neurons in an ANN—if functionality was insufficient for making a correct classification, the organism’s fitness would decrease, and this particular variant of “weights” (interactions in the JAK-STAT signaling pathway) would wash out of the population. In this case, survival of the lineage is how the minimum error is selected.

Next, the process of evolution would begin to “prune” unnecessary connections and/or nodes, pseudogenization being an obvious major mechanism. There are many paths to pseudogenization, but generally, the initial events are nonsense, frameshift, or missense mutations.^47^ A deleterious mutation in a redundant gene does not lead to decreased fitness (selection pressure is relaxed) just as the removal of an unnecessary neuron or a connection in an ANN does not result in a less-accurate model. During the JAK-STAT signaling pathway evolution, gene copies with beneficial mutations were fixed in the populations, while other copies degraded.^46^

Overall, this paradigm for evolution is linked to the aforementioned “double-descent phenomenon,” which suggests that, at least initially, a large increase in complexity (which WGDs provide) is necessary to obtain optimal accuracies.

### Modeling JAK-STAT signaling pathway evolution with ANNs

As suggested above, it is easy to draw parallels between the structure of the JAK-STAT signaling pathway and an ANN. Specifically, we can model an organism’s JAK-STAT signaling pathway as an ANN classifier with a number of inputs representing upstream components, outputs representing downstream components, and an intermediate structure representing the STATs. Subsequently, we can model the evolution of the JAK-STAT signaling pathway under two regimes: (1) varying the number of available inputs and measuring the marginal utility of additional receptors and (2) varying the intermediate structure (layers) and assessing whether a bow-tie architecture is the most efficient one. Thus, we will be modeling the JAK-STAT signaling pathway’s evolution by assessing the marginal utility of added (or pruned) complexity in the different segments of an ANN.

In this case, nodes are analogous to signal transduction elements such as surface-cell receptors or relay molecules like the STAT proteins. A simplified view of a system’s efficiency for the following simulations can be determined via *accuracy per number of nodes* as the production and regulation of each additional signal transduction element has an associated energy cost that must be evaluated against the increase in signal differentiation capacity provided by the additional node.

**Figure 4:**
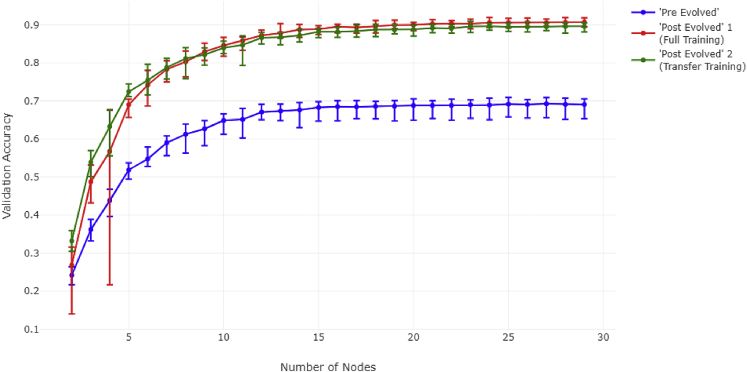
Comparison of ANN performance under different training conditions Error bars are generated over 10 experiments under the specified conditions. Each dataset was sampled using noise level S = 20 (see method details/data generation). “Number of Nodes” references the number of nodes in the intermediary layer. Validation accuracy was calculated on one-third of the data.

We first create a simple simulation to observe the relationship between the complexity of an intermediate structure and model performance (see Figure 4). We also examine the effect additional input information (e.g., more kinds of cytokine receptors) has on that relationship.

We generated synthetic data representatives of 15 classes, with the input vector consisting of 30 elements (see method details/data generation). We set the number of inputs at 30 and the number of outputs at 15 as this approximates in the order of magnitude in the lower bound of the biological reality (the number of cytokines and immune cell “decision”/response alternatives, respectively) in our JAK-STAT example.^40^ We then performed three varieties of ANN simulations: a “pre-evolved” simulation and two “post-evolved” simulations. The pre-evolved simulation was designed to represent a state prior to evolution of more complex elements upstream of STATs (such as more cytokine receptors). The pre-evolved simulation involved training the model on only half of the variables associated with a given class for 600 epochs. The first post-evolved simulation was made to represent a state after the development of the upstream components. This first post-evolved simulation was trained on all variables associated with a given class for 600 epochs. Finally, we create another post-evolved simulation using transfer learning in order to demonstrate that, given enough time, the pre-evolved model can become equivalent to the post-evolved model when introduced to new data (which is a more appropriate analogy for the actual JAK-STAT evolution).

The models used in the experiment are defined in the following way: We specify a fully connected network with three layers—a dense layer with 30 nodes (in the “pre-evolved” model, half the data are zeroed), a dense intermediate layer with a variable number of nodes, and a dense output layer with 15 nodes. The variable number of nodes represents the complexity of the intermediate layer of the network.

The following experiments in this section do not invoke typical ANN pruning methods *sensu stricto* as modern ANN pruning practices utilize a connection-oriented approach to pruning, which has a different kind of analogous biological meaning in the form of, for example, mutations attenuating ligand/receptor binding dynamics, as opposed to pseudogenization. However, in the additional experiment shown in Figure S1, we apply connection-centric pruning methods to demonstrate a rough equivalency between conventional ANN pruning methods and the node-centric approach adopted by us throughout this section.

All models start reaching saturation at approximately the same level of complexity, between 10 and 15 nodes, with no noticeable improvement after 20 nodes. We also note that, given enough time, the “pre-evolved” model can approach the “post-evolved 1” model through transfer learning. Thus, we proceed forward using only the full training model based on this assumption. The results of this simulation show that there exists a point after which a greater complexity of the intermediate structure of an ANN stops yielding improvements in model performance. We observe that this happens regardless of the number of variables (in the input) available to the model. This suggests that doubling the amount of surface-cell receptors would not necessitate the doubling of intermediary communicators.

**Figure 5:**
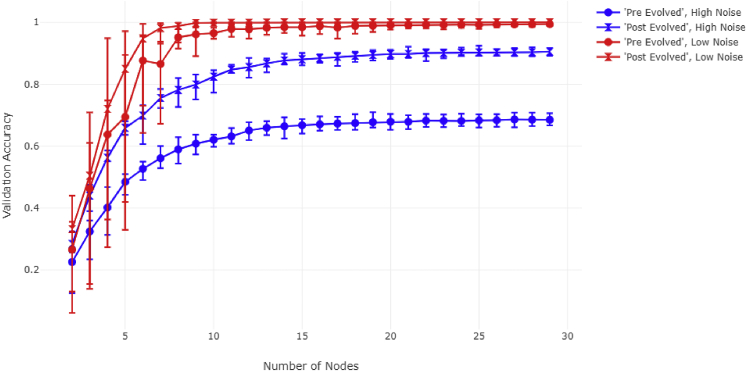
Comparison of ANN performance under high and low noise levels “High noise” corresponds to data generated with S = 10, while “low noise” corresponds to data generated with S = 70. See Figure 4 legend text for further details.

We also wanted to investigate the effect biological noise has on the optimal complexity of a system’s intermediate structure. Using only the previously described “pre-evolved” and the first “post-evolved” model, as well as using synthetic data generated at 7 different noise levels, we carried out simulations to explore how the optimal intermediary structure relates to noise (see Figures 5 and S2).

The noise modification experiment shows that under any tested conditions of separability, the model still reaches peak performance between 10 and 15 nodes. However, we can see that the difference in accuracy between the “post-evolved” and “pre-evolved” states is substantially larger in the case of higher noise levels, demonstrating that access to additional variables is most crucial at higher levels of noise. The biological equivalent of noise could be mutating cytokines, receptors, or both, and thus, evolving more cytokine receptors would lead to higher evolutionary fitness in terms of robustness to noise so long as there is some amount of redundancy in receptor function (i.e., although there was an increase in the number of input elements, the number of output elements remained the same).

**Figure 6:**
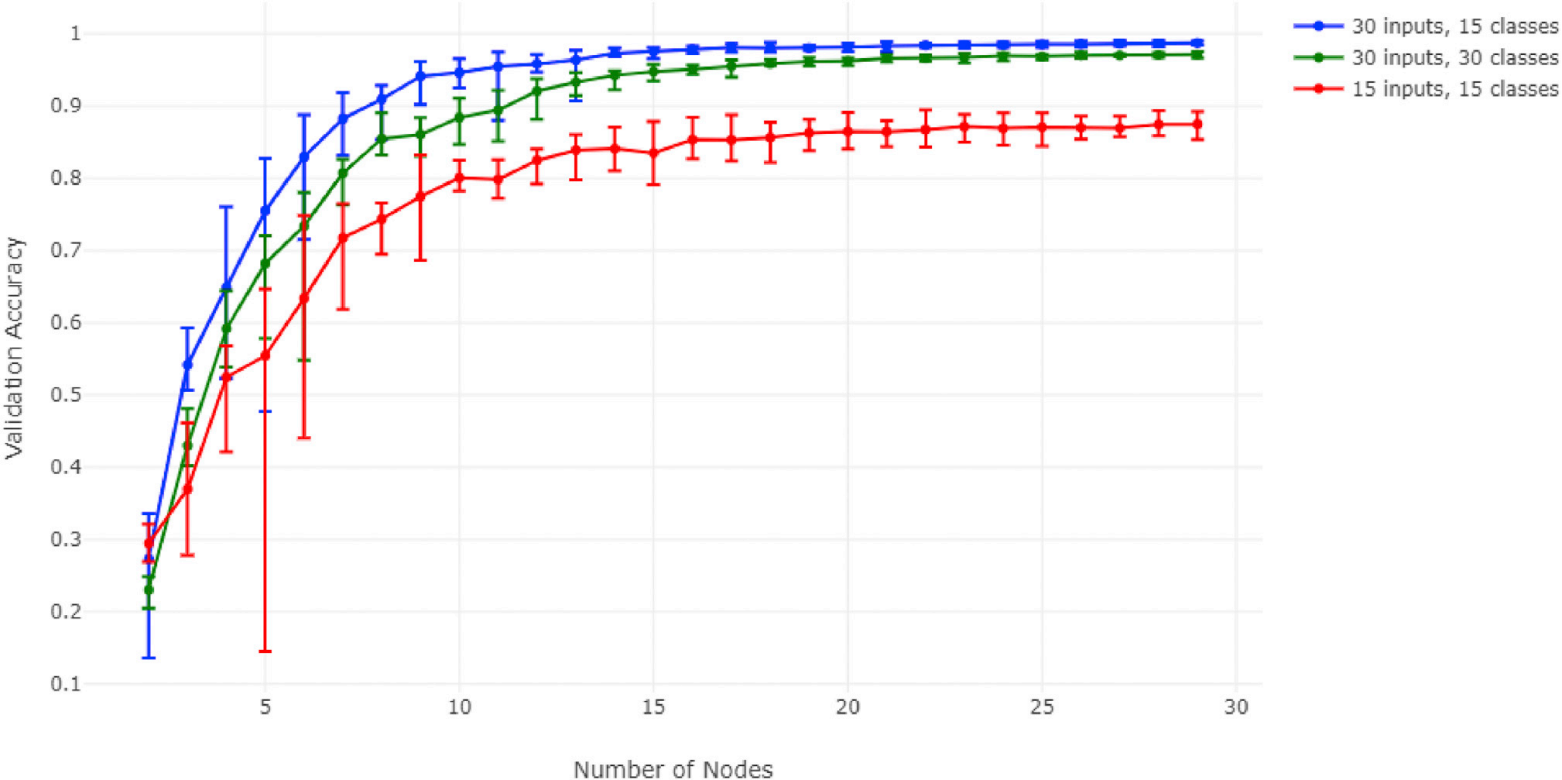
Comparison of ANN performance between models trained with varying numbers of inputs and outputs See Figure 4 legend text for further details.

We also sought to observe the effect of doubling the amount of outputs on network accuracy. We repeated the first experiment in three variations: a 30-input vector with 15 classes, a 15-input vector with 15 classes, and finally a 30-input vector with 30 classes (see Figure 6). All models reach saturation within a range of 5 nodes of each other, with a minimal accuracy difference between the 30/30 case and the 30/15 case.

Interestingly, doubling the number of classes still does not necessitate an increase in intermediary nodes. This means that during evolutionary processes such as WGD, upstream receptors and downstream nuclear interactions can evolve and grow in complexity while building off the same set of communicatory elements in between those two layers. In addition, all models reach saturation around the same number of nodes, meaning it is possible to achieve STATs close to the optimal number prior to evolving all the upstream/downstream components.

**Figure 7:**
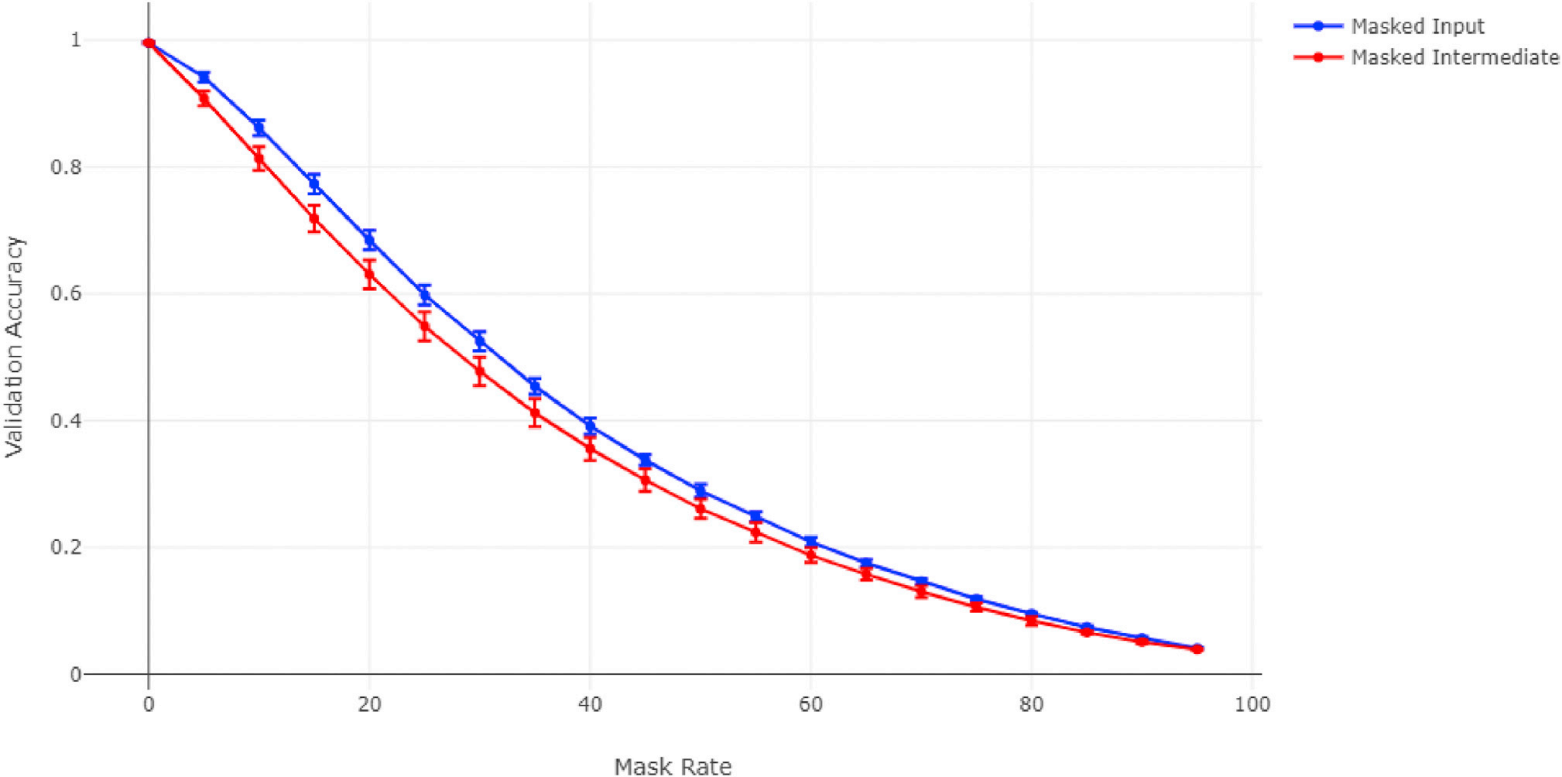
Performance decrease when masking a certain percentage of input or intermediary nodes Validation accuracy was calculated using 80,000 predictions. Mask rate is the percent chance of any node being randomly removed per prediction. The model used in this figure has 20 intermediary nodes.

An essential application of the bow-tie concept is that of information compression. When information transfers from inputs to outputs, the intermediary layers become an embedded structure of that information: This means that the intermediary layers, by necessity, contain all the (“useful”) input information. To demonstrate this in our context, we carried out an experiment based on the 30 input/30 output model with a variable number of intermediary nodes (see Figures 7 and S3). We create a dropout layer after either the input or intermediate layer that has a certain percentage chance to remove any given node during prediction tasks (but not during training). The average accuracy drop over a large number of predictions is then recorded.

The experiment shows that when the same percentage of nodes is masked in either layer, the model decreases in its accuracy by nearly the same amount. This means that the information embedding is virtually equivalent; in fact, the intermediary nodes appear to be even more important due to the processing that has occurred (arguably via decreasing overfitting). Therefore, when studying networks that invoke compression (i.e., they have a bow-tie structure), the intermediary layer tends to have the same (or even more) “useful” information compared to the inputs while simultaneously requiring fewer overall measurements.

## Discussion

### The driving principles behind the construction of modern ANNs are analogous to the driving principles behind evolution and formation of complex biological networks

The impetus behind this study was the two-pronged observation that (1) the bow-tie architecture *in vivo* is similar to that in certain types of ANNs and that (2) well-known evolutionary processes led to the formation of the former, whereas general mathematics and information theory led to the design of the latter. We also observe that the JAK-STAT signaling pathway has a known evolutionary basis that could be analogous to the process (overgrowth followed by pruning) reflective of modern ML practices. Our simulations show how a stable JAK-STAT-like (in its dimensionality and general function) system could evolve alongside its growing upstream and downstream counterparts without necessarily needing a similar increase in its internal complexity.

At their inception, ANNs were modeled after and inspired by the then-current understanding of natural biological NNs, even though their continued development eventually abandoned biological “roots.” It is not uncommon for computer scientists to draw upon biology for inspiration: consider, for example, genetic optimization algorithms likewise modeled after and inspired by the simplified models of gene alleles segregating and propagating in the population—models common in the field of statistical and population genetics.

However, extant complex biological systems’ architectures (such as the ubiquitous “bow tie”) are neither globally optimized nor designed top-down, as ANNs often are. They are, in fact, the results of long evolutionary processes, which are essentially “greedy algorithms” (evolution does not plan ahead, or design top-down), and yet they arrive to similar endpoints as our modern data- and domain-agnostic ML practices.

Therefore, we propose that the emerging techniques in the ML domain could benefit from being informed by known and confirmed evolutionary processes and, inversely, that a mathematical, “first-principles”-style approach to network science could add to our investigations of the evolution of life. This is especially useful for the study of complex biological systems because of the recent advances in our understanding of the network behavior in general.

### Implications for the novel biomarker discovery—prioritization of the compact intermediate layers in the biological networks

While many current research directions focus on the upstream components (i.e., surface-cell receptors) and how they relate to downstream effects (e.g., patient’s response to a drug therapy), it could be more useful to concentrate not on the upstream but rather on the central, fulcrum, components—such as the intermediary layer shown to be at least as informative as the upstream section of the network in Figure 7.

Consequently, in disease states in which a system is not behaving correctly, the more efficient novel biomarker to look for might be situated among the pivotal middle elements, as it will contain any error signal sent from upstream components and any error signal arising from incorrect information processing. In the case of immune signaling, perhaps better prognostic biomarkers could be found by measuring STAT phosphorylation patterns within a cell as opposed to the distributions of surface receptors on a cell. For example, a recent study has found that only six nuclear factor κB (NF-κB) signaling codons were needed to distinguish upstream interactions and inform nearly all downstream processes within a system and that diseased cells had difficulty in distinguishing these signals.^48^ In a parallel example, another recent study has used a neural-network-based method to measure the amount of mutual information between mRNA abundance and Ca^2+^ signaling, finding many individual genes to contain little phenotypic information alone yet being extremely informative when working synergistically.^49^

There are other bow-tie biological systems, such as the MAPK phosphorylation system/pathway (see Figure 1 in the study by García-Hernández et al.^50^), which has many cell surface receptors upstream and groups of transcriptional events (e.g., ERK transcriptional program) downstream. For MAPK-related cancers, biomarkers in the intermediate layer (RAS/RAF/MEK or P38) have proven to be most efficacious, as opposed to the biomarkers in the cell surface receptor (input) or downstream (output) layers^51^. This additionally supports our notion that the best biomarkers are to be discovered at the fulcrum points and layers of the bow-tie-structured biological networks. Interestingly, in the RAS/RAF/MEK/MAPK signaling pathway, the best (so far) biomarkers have been genetic, found through non-flow means (see the study by Williams et al.^51^ and references therein). This suggests that our bow-tie modeling approach can be generalized beyond the flow data and JAK-STAT system, which is a promising avenue for future research.

In general, for any biological system/network, a fruitful venue of purely *in silico* experimentation would be to perform a series of interventions (in different layers and nodes) with a subsequent probabilistic inference propagation in the corresponding BN model, in order to assess the relative impact of the interventions on the downstream eventualities. This promises to present an additional body of evidence in support of our notion (higher salience of more compact intermediate network layers), and such an analysis in the context of JAK-STAT immune signaling in health and cancer is in our immediate-future research plans.

### Conclusion

We believe that a growing interdisciplinary (and cross-pollinating) approach to both evolutionary systems biology and computer science would be mutually beneficial as (1) biologists can lean on new rigorously mathematically founded understanding of optimal information-processing networks to gain insights into the guiding principles of biological networks and (2) computer scientists can look to evolution as a means of getting inspiration from the most robust optimal real-world information-processing system that exists—life.

### Limitations of the study

This study is limited by a lack of measurements of the JAK proteins, as well as a necessarily simplistic simulation scheme (approximating that of a JAK-STAT pathway architecture). We are not measuring dimerization of the STATs at this time. Future research plans include a more complex interrogation of the JAK-STAT pathway under different conditions to experimentally determine if the STAT proteins’ measurements are sufficient for comprehensively capturing various combinations of cytokine stimulus. Furthermore, while the presented results support the notion of concentrating on the intermediate network layers for the biomarker discovery, additional, concrete evidence would be needed to codify this strategy for each specific biological system. Our current work concentrates on constructing and comparing BNs centered around both JAK-STATs (as well as pSMAD (phosphorylated Suppressor of Mothers against Decapentaplegic) 2–3) and cell surface receptors in healthy donors and breast cancer patients and propagating probabilistic inference within the networks, with the goal to demonstrate that changes in the (relatively small number of) pSTATs lead to the more consequential downstream events.

## STAR★Methods

### Key resources table

**Table:** 

REAGENT or RESOURCE	SOURCE	IDENTIFIER
**Antibodies**		

STAT4-pY693 (38/P-STAT4) AF647	BD Biosciences	558137; RRID: AB_397052
CD20 (H1) AF700	BD Biosciences	561171; RRID: AB_10565968
CD14 (HCD14) APCCy7	Biolegend	325620; RRID: AB_830693
STAT6-pY693 (18/p-stat6) V450	BD Biosciences	612601; RRID: AB_399884
PD-L1 (29E.2A3) BV510	Biolegend	329734; RRID: AB_2629580
CD3 (UCHT1) BV570	Biolegend	300436; RRID: AB_2562124
PD-1 (EH12.1) BV605	BD Biosciences	563245; RRID: AB_2738091
RORgT (Q21-559) BV650	BD Biosciences	563424; RRID: AB_2738197
GATA3 (L50-823) BV711	BD Biosciences	565449; RRID: AB_2739242
CD33 (P67.7) BV750	BD Biosciences	746985; RRID: AB_2871764
T-bet (O4-46) BV786	BD Biosciences	564141; RRID: AB_2738615
CD45RA (HI100) BUV395	BD Biosciences	740298; RRID: AB_2740037
Live/Dead Blue	Invitrogen	L23105
CD4 (SK3) BUV563	BD Biosciences	612912; RRID: AB_2870197
CD16 (3GB) BUV737	BD Biosciences	612786; RRID: AB_2833077
CD8 (SK1) BUV805	BD Biosciences	612889; RRID: AB_2833078
STAT3-pY705 (4/P-STAT3) AF488	BD Biosciences	557814; RRID: AB_647098
STAT1-pY701 (4a) PerCP-Cy5.5	BD Biosciences	560113; RRID: AB_1645550
Smad 2-pS465/pS467/Smad 3 -pS423/pS425 (O72-670) PE	BD Biosciences	562586; RRID: AB_11151915
FOXP3 (259D/C7) PE-CF594	BD Biosciences	562421; RRID: AB_11153143
STAT5-pY694 (47) PECy7	BD Biosciences	560117; RRID: AB_1645546

**Chemicals, peptides, and recombinant proteins**		

Recombinant Human IFN-γ	PeproTech	300–02
Recombinant Human IL-10	PeproTech	200–10
Recombinant Human IL-2	PeproTech	200–02
Recombinant Human 1L-12p70 (HEK293 derived)	PeproTech	200–12H
Recombinant Human IL-4	PeproTech	200–04
Recombinant Human TGF-β1 (CHO derived)	PeproTech	100–21C
Recombinant Human IL-6	PeproTech	200–06

**Software and algoirthms**		

BNOmics	City of Hope National Medical Center	https://bitbucket.org/77D/bnomics/src/master/
Python version 3.8	Python Software Foundation	https://www.python.org/
Tensorflow v2.0.0	Google	https://www.tensorflow.org/
NumPy v1.23.0	NumPy	https://numpy.org/
Data generation, ANNs, ANN training, simulation experiments	This communication	This communication

### Resource availability

#### Lead contact

Further information and requests for code and data should be directed to and will be fulfilled by the lead contact Seth Hilliard (shilliard@coh.org).

#### Materials availability

This study did not generate any materials.

### Method details

#### Biological data generation

PBMCs from four estrogen receptor (ER)+ breast cancer patients (collected at diagnosis, before treatment) and seven age-matched female healthy donors (IRB protocol #11273) were isolated using Ficoll-Paque Plus (GE-Heaslthcare), aliquoted and frozen in liquid nitrogen until experiment. On the day of experiment, frozen PBMCs were thawed and rested in complete medium (RPMI 1640 (Gibco) + 10%FBS) at 37°C for 2-3h before staining with 1:1000 diluted Fixable Blue Live/Dead dye (Invitrogen) in PBS at 37°C for 15min. After washing, cells were resuspended into 10^6^–10^7^ cells/mL in complete medium and aliquoted into 200uL/well in 96-well deep-well plate, then rested at 37°C for at least 15min. PBMCs were stimulated by adding 50ul of single cytokines to their final concentrations of IFN-γ (1 ng/mL); IFN-γ (50 ng/mL); IL-6 (1 ng/mL); IL-6 (50 ng/mL); IL-10 (50 ng/mL); IL-12 (100 ng/mL); IL-2 (50 ng/mL); IL-4 (50 ng/mL); TGF-β (50 ng/mL); or in combination of cytokines IFN-γ (1 ng/mL) + IL-6 (1 ng/mL); IFN-γ (50 ng/mL) + IL-6 (1 ng/mL); IFN-γ (1 ng/mL) + IL-6 (50 ng/mL); IFN-γ (50 ng/mL) + IL-6 (50 ng/mL) for 15 -or 60 min at 37°C. After stimulation, cells were fixed by adding 25uL 16% PFA (Electron Microscopy Sciences) at RT for 10min then washed once using PBS by spinning down at 1258g for 5–10min. After washing, cells were resuspended using 500uL pre-chilled 100% methanol per well, kept in 4 °C for 30min then transferred to −80 °C overnight. On the second day, cells were washed three times using FACS buffer (5% FBS, 0.5% Sodium Azide in PBS). Cells were incubated with 5% Fc blocker (Biolegend) in FACS buffer at RT for 5–10min then stained using antibody cocktail at RT for 45min. Cells were washed once using FACS buffer then analyzed using Cytek Aurora. Data were handled using FlowJo software. Characteristics of four breast cancer patients are detailed in the table below. Additional data was collected from seven healthy controls.

**Table:** 

Patient ID	Age	ER/PR/Her2	Grade	Ki67 (%)	T	N	Stage
05	46	+/+/−	2	20	1	2	III
06	61	+/+/−	3	20	2	0	II
07	47	+/+/−	1	1–5	1	0	I
08	71	+/+/−	1	5–10	1	1	II

#### Bayesian network (BN) modeling

BNs were constructed with BNOmics^52^ using a hybrid constraint-based + search-and-score algorithm with 20 restarts and MDL scoring function. Continuous variables were discretized in 8 bins using MaxEnt. Edge strengths in the networks are proportional to the marginal likelihood ratios, given the data, of the model with the edge to the model without. See^38^^,^^52^ for further details.

#### Data generation

The data generated for this work is a set of vectors sampled from a multivariate Gaussian distribution, with a given distribution representing a specific class that we try to predict. These distributions are either 15 dimensional or 30 dimensional, with each element randomly being one of two values: 0.3 (representing “negative” signals) or 0.7 (representing “positive” signals). We then generate a set of training data from each class by repeatedly sampling from the class but adding some amount of noise to each element. This is calculated by multiplying the vector by the identity matrix divided by the noise value S. Each model was trained and tested using 1000 data elements per class, with 33% of the data used for validation. Thus, the input data is generated using a multivariate Gaussian with a mean defined separately for every class and with a constant covariance matrix across the classes.

#### Model architecture and training

We apply a ReLU activation^53^ after the first two layers and a Softmax^54^ after the output layer. The variable number of nodes represents the complexity of the intermediate structure of the network. The model is retrained on the same data every time the number of intermediate nodes are adjusted.

## Data Availability

BNOmics (software for high-dimensional large-scale Bayesian network modeling) is available at https://bitbucket.org/77D/bnomics/src/master/. Additional data and code (for data generation and simulation experiments) is included with this communication, in supplemental information. All intermediate/auxiliary datasets will be made available by the authors, without undue reservation, to any qualified researcher.
