# Supplementary material for: Bow-tie architectures in biological and artificial neural networks: Implications for network evolution and assay design
Source: iScience. 2023 Jan 25;26(2):106041. doi: 10.1016/j.isci.2023.106041 (PMC9929672; doi:10.1016/j.isci.2023.106041)
Supplement: Document S1. Figures S1–S3 [file mmc1.pdf]

iScience, Volume *26*

Supplemental information

Bow-tie architectures in biological and artificial neural networks: Implications for network evolution and assay design

Seth Hilliard, Karen Mosoyan, Sergio Branciamore, Grigoriy Gogoshin, Alvin Zhang, Diana L. Simons, Russell C. Rockne, Peter P. Lee, and Andrei S. Rodin

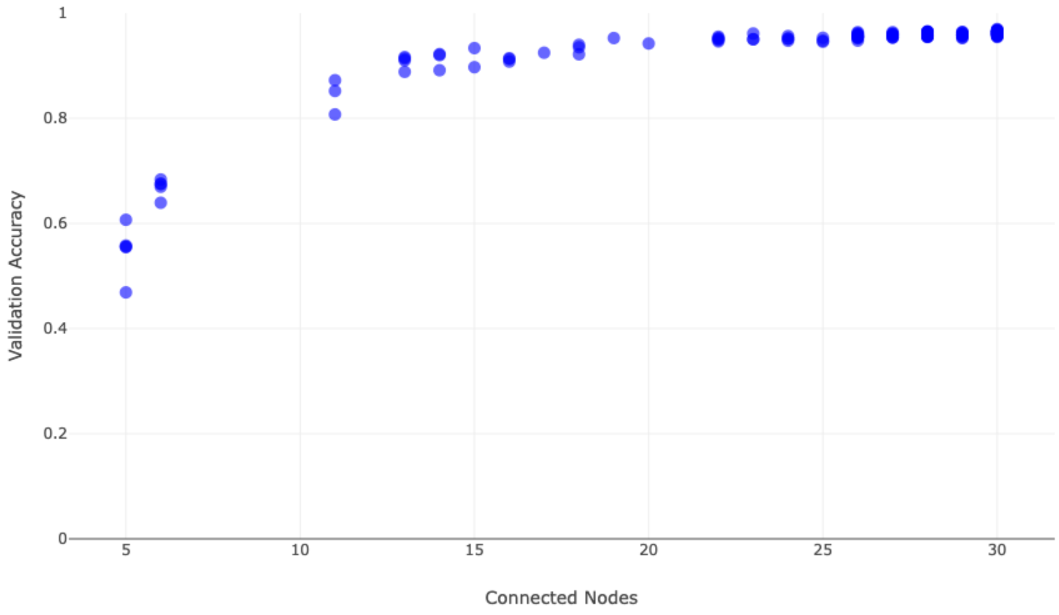

Supplemental Figure 1. Validation Accuracy of a pruned model against the number of connected nodes remaining in the intermediate layer, related to Figure 4.

Each data point was generated at one of the following pruning rates: 0.2, 0.5, 0.6, 0.65, 0.7, 0.75, 0.8, 0.85, 0.9, 0.925, 0.95, 0.975, 0.985, 0.995. 10 models per pruning rate were trained following the 30 input - 30 class model used in Fig. 6 and pruned. The pruning rate does not directly dictate the number of pruned nodes, therefore a certain number of remaining nodes may be achieved with different pruning rates. THe pruning method prioritizes weights with lowest magnitudes first.

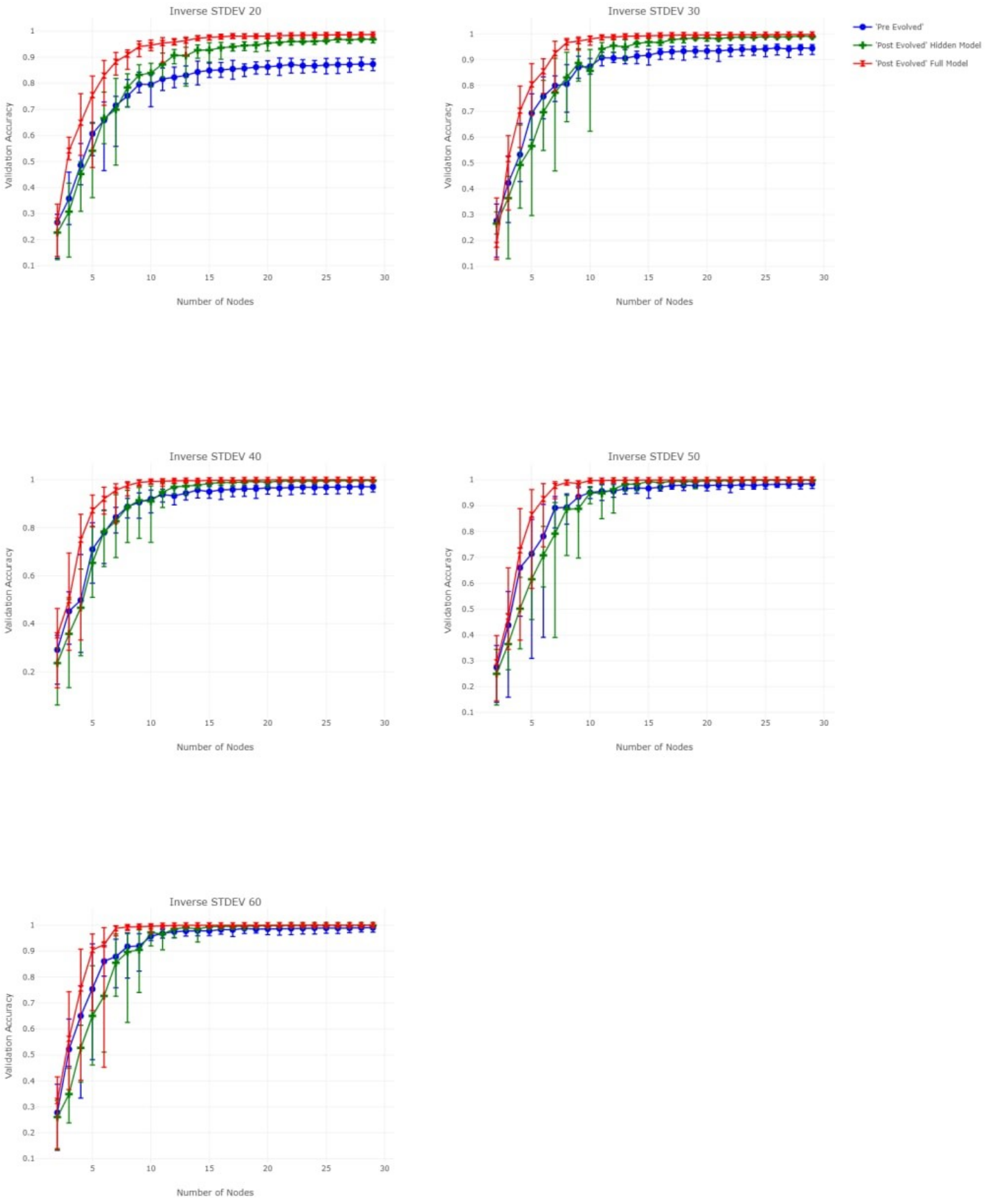

Supplemental Figure 2. Comparison of ANN performance trained in the 'pre-evolved' state (hidden training, blue) and the 'post-evolved' states with continued learning (transer training, yellow; full training, red) at various noise levels, related to Figure 5.

Smaller values of inv_std corresponding to higher amounts of noise. Inv_std takes the value os 20, 30, 40, 50 and 60 going from legt to right and top to bottom.

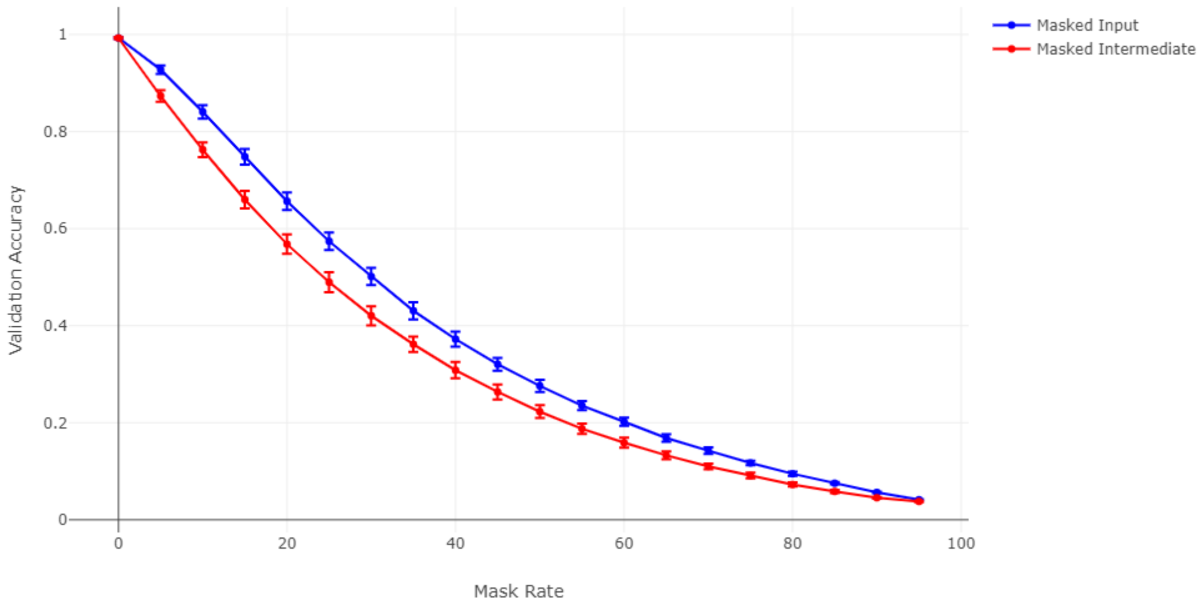

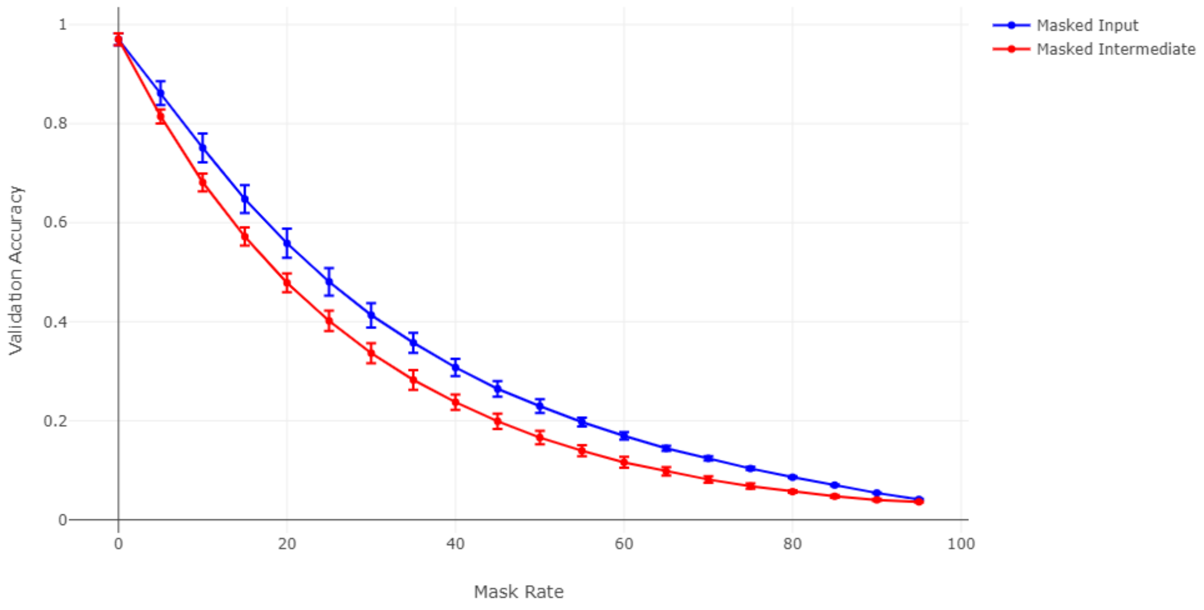

Supplemental Figure 3. Mask rate experiment using 15 intermediary nodes (top) and 10 intermediary nodes (bottom), related to Figure 7.

Validation accuracy was calculated using 80,000 predictions. Mask rate is the percent chance of any node being randomly removed.
